# Effects of Propofol *Versus* Sevoflurane on Postoperative Breast Cancer Prognosis: A Narrative Review

**DOI:** 10.3389/fonc.2021.793093

**Published:** 2022-01-20

**Authors:** Panpan Fang, Jiaqi Zhou, Zhengyuan Xia, Yao Lu, Xuesheng Liu

**Affiliations:** ^1^ Department of Anesthesiology, The First Affiliated Hospital of Anhui Medical University, Hefei, China; ^2^ Department of Anesthesiology, Affiliated Hospital of Guangdong Medical University, Zhanjiang, China; ^3^ State Key Laboratory of Pharmaceutical Biotechnology, Department of Medicine, The University of Hong Kong, Hong Kong, Hong Kong SAR, China

**Keywords:** propofol, sevoflurane, breast cancer, metastasis, recurrence, long-term prognosis

## Abstract

Perioperative interventions produce substantial biologic perturbations which are associated with the risk of recurrence after cancer surgery. The changes of tumor microenvironment caused by anesthetic drugs received increasing attention. Till now, it’s still unclear whether or not anesthetic drugs may exert positive or negative impact on cancer outcomes after surgery. Breast cancer is the most common tumor and the leading cause of cancer deaths in women. Propofol and sevoflurane are respectively the most commonly used intravenous and inhaled anesthetics. Debates regarding which of the two most commonly used anesthetics may relatively contribute to the recurrence and metastasis vulnerability of breast cancer postoperatively remain. This review aimed to provide a comprehensive view about the effect of propofol versus sevoflurane on the prognosis of breast cancer obtained from pre-clinical studies and clinical studies. Laboratory and animal studies have demonstrated that sevoflurane may enhance the recurrence and metastasis of breast cancer, while propofol is more likely to reduce the activity of breast cancer cells by attenuating the suppression of the immune system, promoting tumor cells apoptosis, and through other direct anti-tumor effects. However, retrospective clinical studies have shown contradictory results about the effects of propofol and sevoflurane on long-term survival in breast cancer patients. Furthermore, recent prospective studies did not identify significant differences between propofol and sevoflurane in breast cancer metastasis and recurrence. Therefore, more preclinical studies and randomized controlled studies are needed to guide the choice of anesthetics for breast cancer patients.

## Introduction

Breast cancer is the most commonly diagnosed malignant tumor and the leading cause of cancer-related death among females. It was estimated that there were more than 2 million new cases and 0.63 million cancer related deaths worldwide in the single year of 2018 (1). Surgical removal of the tumor is the foremost treatment strategy for breast cancer (2). However, the scattered micro-metastases and tumor cells after surgery inevitably formed residual disease. Due to the residual disease, a considerable percentage (ranging from 10 to 41%) of surgical cancer patients will suffer from the recurrence of cancer at five years postoperatively depending on different tumor grades and tumor-node-metastasis staging (3). Whether tumor recurs or metastasizes depends on the balance between the immune capacity of the host and the progression of residual disease. The mortality of breast cancer was attributable to the recurrence and distant organ metastasis and the five-year survival rate was ranging from 69.5% to 93.8% (4, 5). The high recurrence rate after surgery questions whether or not there are any perioperative measures that may shift the balance towards host defense to reduce the risk of disease recurrence.

There have been increasing concerns that perioperative substantial biologic perturbations will increase the risk of recurrence after cancer surgery (6). On the one hand, tissue trauma and perioperative stress responses are associated with increases in proinflammatory cytokines, inflammatory factors (7) and stress hormones (8). These might promote the growth of residual tumor cells and increase the vulnerability to cancer recurrence by inducing transient suppression of cell-mediated immunity (9) and by releasing proangiogenic factors such as vascular endothelial growth factor (VEGF) (10). On the other hand, the changes of tumor microenvironment caused by anesthetic drugs is also an area of particular concern (11). Till now, it’s still unclear whether anesthetic drugs may exert positive or negative effect on cancer outcomes (12). Propofol and sevoflurane are respectively the most commonly used intravenous and inhaled anesthetics. These two anesthetics have different effects on tumor cells and immune function (13). Which one contributes to the postoperative recurrence and metastasis vulnerability has received increasing attentions (14–16).

This review aimed to compare the effects of propofol versus sevoflurane on immune system, breast cancer cells and patient long-term outcomes observed from pre-clinical studies and clinical studies. We searched PubMed database with search terms (“propofol” or “sevoflurane”) and (“breast cancer” or “breast tumor”) on Sept. 30, 2021 to obtain the literatures in this review, and only the articles written in English were included.

## Immune Pathogenesis of Tumorigenesis

The innate and adaptive immune system are vital to the body’s surveillance against cancer. The complex processes of cancer cell invasion and metastasis are involved in the “elimination” phase, “equilibrium” state and “escape” phase. During the “elimination” phase, the natural killer (NK) cells, CD4^+^Th1, CD8^+^CTL (cytotoxic T lymphocyte), and cytokines including tumor necrosis factor-α (TNF-α), interferon-α, interferon-β, interferon-γ and interleukin-12(IL-12) are the primary factors to recognize and eliminate cancer cells (17). If the cancer cells have escaped elimination and entered into “equilibrium” state, the adaptive immune response began to play a key role in preventing cancer cells from further growth. When the cancer cells enter into the final “escape” phase, the immune control of the host is usually insufficient to inhibit the growth of tumor cells, leading to apparent growth ultimately.

**Figure 1:**
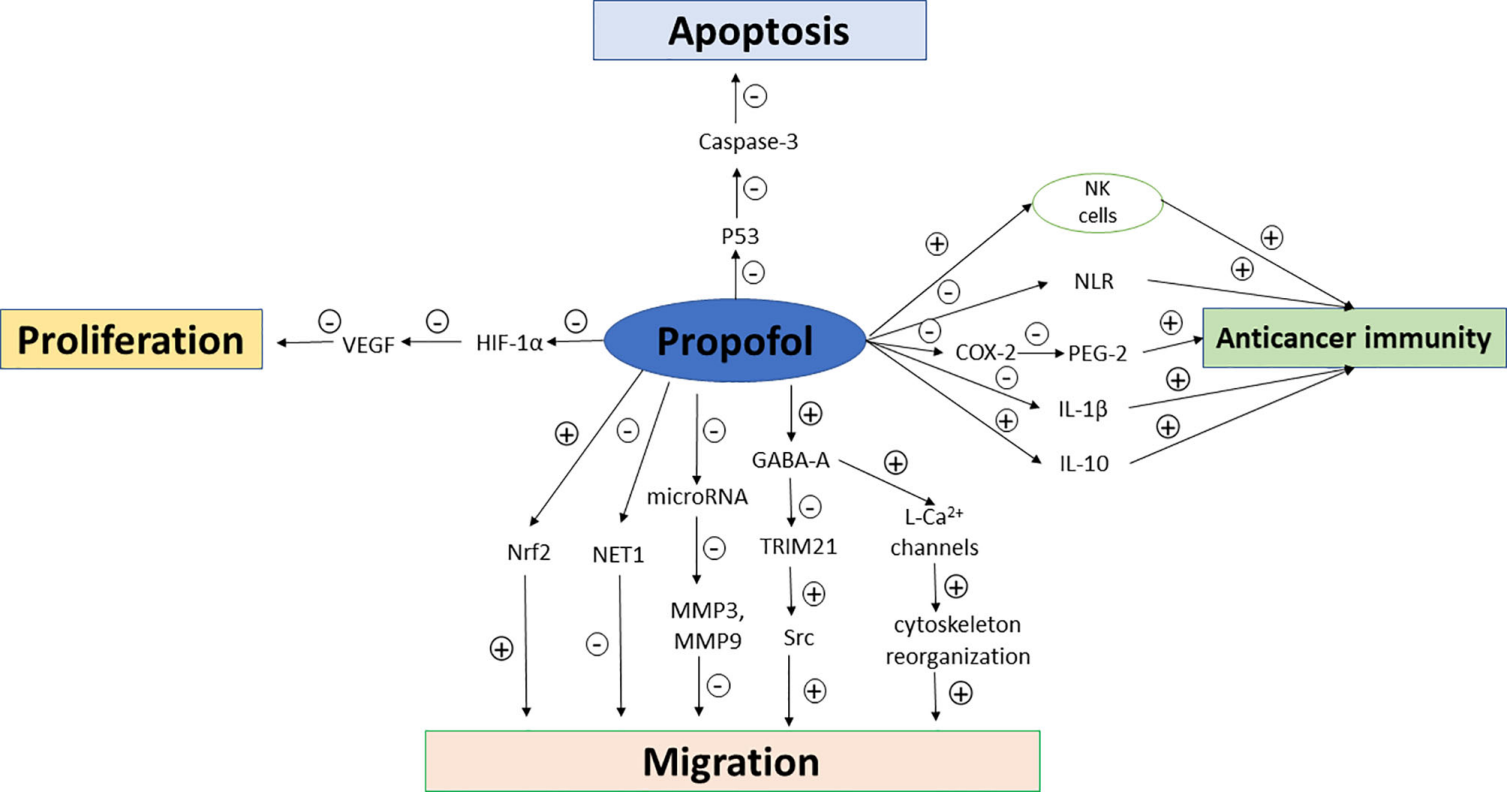
Mechanisms of propofol on anticancer immunity, breast cancer cell proliferation, migration and apoptosis. NK cells, natural killer cells; NLR, neutrophil–lymphocyte ratio; COX-2, cyclooxygenase-2; VEGF, vascular endothelial growth factor; PEG-2, prostaglandin E2; GABA, gamma aminobutyric acid; MMP, Matrix metalloproteinases; HIF-1α, hypoxia inducible factor-1α; TRIM21, tripartite motif 21; Src, non-receptor tyrosine kinase; Nrf2, nuclear factor E2-related factor-2.

**Figure 2:**
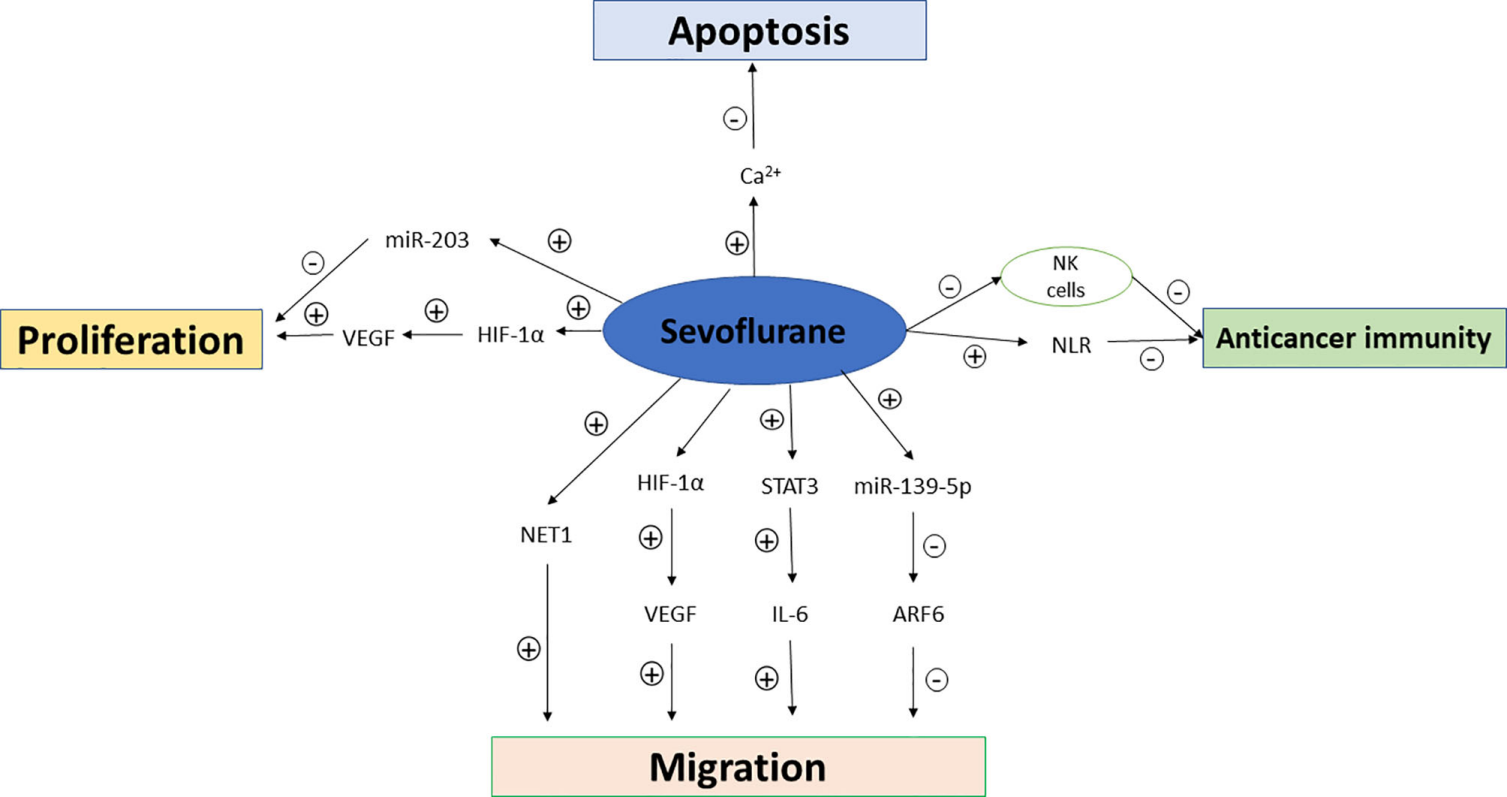
Mechanisms of sevoflurane on anticancer immunity, breast cancer cell proliferation, migration and apoptosis. NK cells, natural killer cells; NLR, neutrophil–lymphocyte ratio; VEGF, vascular endothelial growth factor; HIF-1α, hypoxia inducible factor-1α; NET1, Neuroepithelial Cell Transforming Gene; STAT3, signal transducer and activator of transcription 3; ARF6, ADP-ribosylation factor 6.

In addition to the host’s anti-tumor immunity, tumor cells also produce mediators that fight against host immunity in order to promote their own growth. The cytokines such as VEGF and transforming growth factor-β (TGF-β) which are produced by tumor cells can induce immunosuppressive effects (18, 19). Some inflammatory factors and proinflammatory cytokines including interleukin-6 (IL-6), IL-1β, and prostaglandin E2 (PGE-2) also promote tumor growth. The effects of sevoflurane and propofol on postoperative inflammatory cytokine release were compared in patients undergoing other major surgeries (20, 21), but not in those undergoing breast cancer surgeries so far. Furthermore, regulator T cells, tumor-associated macrophages and myeloid-derived suppressor cells (MDSCs) recruited by cancer cells also favor tumor progression (22). Propofol attenuated the decrease in CD39 and CD73 in circulating CD4+ T cells compared to sevoflurane-based anesthesia in patients undergoing open heart surgeries (23), while similar comparative studies have not been reported in breast cancer patients despite that circulating regulatory T cells has been recently reported to be significantly increased in breast cancer patients which may impact on the stage and histological type of breast cancer (24). The possible mechanisms of propofol and sevoflurane on anticancer immunity, breast cancer cell proliferation, migration and apoptosis are summarized in **Figures 1** and **2**.

## Effects of Sevoflurane and Propofol on Cancer Immune System

Sevoflurane is the most popular volatile anesthetics due to the advantages of fast induction, small respiratory tract stimulation, fast absorption and clearance, less circulation disturbance. Propofol, a kind of alkyl acid short acting anesthetics, is the most commonly used intravenous anesthetic. Laboratory researches have shown that propofol-based intravenous anesthesia and sevoflurane-based inhalation anesthesia may have different effects on breast cancer immune microenvironment.

NK cells, CD8^+^ CTL and CD4^+^ Th1 cells are the important weapons to fight against cancer cells (22). By contrary, MDSCs, tumor-associated macrophages and CD4^+^ Th2 cells promote tumor formation and growth by inhibiting the anti-cancer immune response. Ample evidences support that propofol can enhance anti-tumor immunity by increasing the activity of anti-tumor immune cells. NK cells, a subpopulation of large granular lymphocytes, play an important role in anti-tumor immunity due to direct recognition and lysis of cancer cells (25, 26). Reduction in NK cell numbers and activities make the host prone to promote tumor formation or tumor metastasis (27, 28). Melamed et al. compared the effects of different anesthetics on NK cell activity and tumor metastasis. They found that ketamine, thiopental and halothane but not propofol significantly reduced NK cell activity and promoted MADB106 breast cancer cell metastases (29). Inada and colleagues demonstrated that propofol increased the production of interferon-gamma (IFN-γ) *via* activating NK cells subsequent to the suppression of thioglycollate-elicited murine peritoneal macrophages (30). And, this team further found that the aforementioned effect of propofol was achieved through inhibiting cyclo-oxygenase activity in human monocytic cell line THP-1 (31). A pilot study from Ireland collected the serum from patients who received propofol-paravertebral block (PPA) or sevoflurane-opioid anesthetic techniques, and co-cultured the serum with breast cancer cells (32). This study showed that the cytotoxicity of NK cells and breast cancer cells apoptosis increased in the serum from patients who received PPA anesthesia technique. The same research team investigated the effect of PPA *vs.* sevoflurane-opioid analgesia on immune cell infiltration in breast cancer tissue, and they also found increased levels of NK cells and T helper cell infiltration into breast cancer tissue in the PPA group (33). A prospective randomized study assigned breast cancer patients to receive propofol anesthesia with ketorolac analgesia and sevoflurane anesthesia with fentanyl analgesia, and the results showed that NK cell cytotoxicity was increased in propofol with ketorolac group, but decreased in the sevoflurane with fentanyl group (34). On the other hand, an *in vitro* study showed that there was no difference in NK cell count, cytotoxic T lymphocyte counts and breast cancer cell apoptosis rate between propofol and sevoflurane groups (35).

Both increased inflammation and reduced cell-mediated immunity contribute to an increase in neutrophil–lymphocyte ratio (NLR) (36). Increased NLR and platelet–lymphocyte ratio (PLR) are related to increased risk of breast cancer recurrence and metastasis (37, 38). Eochagáin et al. performed a subgroup analysis of a randomized study, they found that propofol-paravertebral anesthesia during breast cancer surgery was associated with less increase of NLR when compared with sevoflurane-opioid anesthesia (39). Cluster of differentiation (CD) enzymes on regulatory T cells have immunosuppressive effects. CD39 and CD73 on regulatory T cells have been confirmed to play important roles in promoting cancer recurrence and metastasis due to the impairment of the activities of NK cells and CTL (40, 41). A randomized trail compared the differences between propofol and sevoflurane in CD39 and CD73 expression on regulatory T cells. This study found that there was no difference in the expression of CD39 and CD73 between propofol and sevoflurane anesthesia groups at 1 and 24 hours postoperatively (42). MDSCs play a key role in immune suppression, tumor angiogenesis and tumor metastases in cancer patients (43). MDSC consists of polymorphonuclear MDSC (PMN-MDSC) and monocytic MDSC (M-MDSC). PMN-MDSC are morphologically and phenotypically similar to neutrophils while M-MDSC are similar to monocytes morphologically (44). Yan et al. compared the MDSC expression in breast cancer patients who received sevoflurane-based anesthesia or propofol-based anesthesia. They found that there was no significant difference in MDSC expression between these two groups, whereas MDSC expression and the subtype of MDSC were correlated to tumor stages (45). Most studies have shown that propofol anesthesia increased NK cells cytotoxicity, NLR and PLR as compared with sevoflurane. However, a few studies showed no difference in in between propofol and sevoflurane anesthesia regarding the impacts on T lymphocyte cytotoxicity and MDSC expression.

## Effects of Sevoflurane and Propofol on Functions of Breast Cancer Cells

Breast cancer cells have about 21 diverse histological subtypes. According to different presences of estrogen receptor (ER), progesterone receptor (PR) and human epidermal growth factor receptor 2 (HER2), the diverse subtypes are stratified into four major molecular subtypes namely triple negative breast cancer cell, HER2 overexpressing breast cancer cell, Luminal A breast cancer cell and Luminal B breast cancer cell. Triple negative breast cancer cell is ER-/PR-/HER2-; HER2 overexpressing breast cancer cell is ER-/PR-/HER2+; Luminal B breast cancer cell is ER+ and/or PR+/HER2+; Luminal A breast cancer cell is ER+ and/or PR+/HER2-. In recent years, the potential impact of different general anesthetics on tumor prognosis has garnered particular attention. Different breast cancer cell lines were cultured *in vitro* to investigate the effect of anesthetics on breast cancer cell proliferation, migration and apoptosis (46).

An *in vitro* study investigated the effect of sevoflurane on breast cancer cell proliferation, migration and invasion (47). In this study, MDA-MB-231 ER– and MCF7 ER+ breast cancer cells were incubated with sevoflurane at different concentrations. It was found that sevoflurane increased the proliferation and migration in both breast cancer cell lines, however, the increased invasion was only observed in ER+ cells. In another *in vitro* study, the authors co-cultured MDA-MB-231 ER- cell with the serum from patients who received either PPA or sevoflurane-opioid anesthetic techniques. The authors found that the proliferation of cancer cells was reduced in PPA group compared with sevoflurane-opioid group, while there was no significant difference in migration between two groups (48). Apoptosis of tumor cells is also an important factor that affects breast cancer recurrence and metastasis. A study showed that the apoptosis rate of MDA-MB-231 ER– cells was higher in cells exposed to human serum from patients who received PPA than in cells exposed to human serum from patients who received sevoflurane-opioid anesthesia (49).

Activation of specific gene during the perioperative period may accelerate tumor recurrence and metastasis. Neuroepithelial Cell Transforming Gene 1(NET1) has been identified to have the property of promoting tumor cells migration (50), and has been used as potential prognostic marker for patients (51). An *in vitro* study showed that sevoflurane treatments increased the NET1 gene expression in metastatic canine tubular adenocarcinoma cells at the concentration of 4mM (52). Patricija et al. demonstrated that propofol reduced both MCE7 ER+ and MDA-MB-231 ER-breast cancer cell migration by the down-regulation of NET1 expression (53). In addition, hypoxia inducible factor-1α (HIF-1α) is a key regulator in hypoxia inducing tumor growth. HIF-1αinduces the secretion of angiogenic factors such as VEGF and angiogenic 2 (54, 55). Therefore, up-regulated expression of HIF-1α has been shown to augment tumor angiogenesis, promote tumor cell proliferation (56) and has been associated with poor prognosis. A recent study also demonstrated that HIF-1α signaling selectively enhanced breast cancer cell proliferation in the brain (57). HIF-1 also plays an important role in breast cancer cell metastasis by regulating multiple key steps of metastasis, such as epithelial-mesenchymal transition, metastatic niche formation, invasion, and extravasation (58). An experimental study showed that 2 mM sevoflurane exposure 72h increased the viability, proliferation and aggressive of triple negative breast cancer and increased HIF-1 expression (59). There are few researches investigating the effect of propofol on HIF-1αin breast cancer cells. However, propofol has been identified to inhibit HIF-1αactivation induced by hypoxia in prostate cancer which may shed light to the mechanism of propofol in breast cancer (60).

## Effects of Sevoflurane and Propofol on Microenvironments of Breast Cancer Cells

Matrix metalloproteinases (MMPs) provide a favorable microenvironment for tumorigenesis by digesting extracellular matrix components. MMPs also release pro-cancer factors from the extracellular matrix to promote tumor cell migration (61). The levels of MMPs were higher in cancer patients (62, 63). Patients undergoing primary breast cancer surgery who received propofol/paravertebral anesthesia had less elevated MMP-3 and MMP-9 as compared with those who received sevoflurane based anesthesia during primary breast cancer surgery (64). At the same time, propofol has been demonstrated to significantly decrease IL-1β, but significantly increase IL-10 postoperatively as compared with sevoflurane (64). Conversely, sevoflurane has been reported to lead to more lung metastasis with higher level of serum IL-6 *via* activating STAT3 and infiltrating CD11b+ cells as compared to propofol (65). General anesthetics may also influence tumor cells by changing angiogenic factor. VEGF and TGF-β are secreted by tumor cells to help themselves grow and metastasize (66, 67). A prospective randomized study allocated breast cancer patients to sevoflurane group and propofol group respectively, and this study showed that serum VEGF concentrations were significantly higher after surgery in the sevoflurane group than in the propofol group, however, the serum VEGF concentrations kept unchanged in propofol group, and the concentrations of TGF-β did not significantly differ between sevoflurane and propofol groups both before and after surgery (68).

Ca^2+^, a kind of second messenger, plays a key role in numerous cellular processes including cell proliferation and apoptosis (69). Abnormal Ca^2+^ signaling pathways and Ca^2+^ transport proteins are associated with breast tumor tumorigenesis (70). A study investigated the effects of sevoflurane versus propofol on three kinds of breast cells and Ca^2+^ homeostasis. This study showed that sevoflurane at the concentration of 2% for 6 hours duration increased the survival of both ER- and ER+ breast cancer cells *in vitro* and chelation of cytosolic Ca^2+^ significantly decreased the survival of breast cancer cells (71). Therefore, it can be inferred that breast cancer cells need more cytoplasmic Ca^2+^ for survival, and sevoflurane may increase breast cancer cells survival *via* modulating intracellular Ca^2+^ homeostasis. Indeed, in a mouse model of breast cancer (72), regulation of the microRNA-129-1-3p-mediated calcium signaling pathway has been shown to restrain the growth of breast cancer cells. MicroRNAs are noncoding RNA molecules which participate in post-transcriptional gene regulation. There are more than 1500 miRNA molecules in human body, and miRNAs play critical roles in various cell biology (73, 74). Variations of miRNA expression may affect cancer cell activity and lead to tumor recurrence and metastasis (75, 76). Studies have reported that sevoflurane suppresses breast cancer cell proliferation by upregulating miR-203 (77). Sevoflurane suppressed the invasion, migration, and epithelial-mesenchymal transition of breast cancer cells through downregulating the abundance of ARF6 by upregulating miR-139-5p (78). Propofol has also been reported to affect miRNA and reduce matrix metalloproteinase expression to change anti-cancer microenvironment (79).

It should be noted that there are also studies which showed that propofol had pro-tumor effects in breast cancer. Garib et al. observed that the percentage of MDA-MB-468 cells migration, the velocity and distance of migration were increased in a dose-dependent manner when the breast cancer cells were incubated with various concentrations of propofol (80). They further confirmed that propofol increased breast cancer cell migration through activating gamma aminobutyric acid A (GABA-A) receptor (81), and the process was mediated by increased intracellular calcium *via* L-type calcium channels and the actin cytoskeleton reorganization (81). In another *in vitro* study, MDA-MB-231 cells were treated with propofol at 2-10 ug/ml for 1-12 hours (82). The authors also found that propofol increased breast cancer cells proliferation and migration in a dose- and time-dependent manner. The authors further found that the increased proliferation may be mediated through downregulation of p53 protein, while the promotion of migration may be mediated *via* the activation of the Nrf2 pathway (82). A recent study also demonstrated that propofol promoted tumor metastasis by activating GABA-A receptor, downregulating TRIM21 expression, and upregulating Src (a protein associated with cell adhesion) expression (83). It should be noted that there may be several factors resulting in the inconsistent effects of propofol on breast cancer cells. First of all, different breast cancer cells with different biological characteristics may contribute to the discrepancy. Secondly, the concentration and duration of propofol exposure were variant in different researches.

## Long-Term Prognosis of Patients

The effects of anesthetics on tumor immune microenvironment and tumor cells have been documented in well-designed laboratory and animal studies. However, the results of pre-clinical studies should be interpreted with caution. The clinical studies in human are also needed to investigate the association between anesthetics and long-term cancer outcome.

## Retrospective Studies

The currently available retrospective studies comparing propofol with inhalation anesthetics on long-term prognosis of breast cancer surgery were summarized in **Table 1**. The first one was published in 2014 by Enlund and colleagues (84). The data in this study was from a single hospital of Sweden between January 1998 to 31 March 2010. This study reviewed 1837 breast cancer patients with 620 patients in propofol group and 1217 patients in sevoflurane group. The 1-year survival rate were 99% in propofol group and 96% in sevoflurane group respectively, and the difference was 3% (p<0.001). However, the difference of 5-year survival rate between these two groups was 2% (84% in propofol group *versus* 82% in sevoflurane group) with no statistical significance. Then, a retrospective study from Korea compared the recurrence-free survival and overall survival between propofol and sevoflurane groups in patients after modified radical mastectomy (85). This study included 325 cases with 173 patients in propofol group and 152 patients in sevoflurane group. The 5-year survival rate was comparable between the two groups. However, there was a lower cancer recurrence rate in propofol group (p=0.037), and the hazard ratio of recurrence was 0.55. A larger sample size retrospective cohort study from the United Kingdom enrolled 11395 patients undergoing mixed cancer surgery. After propensity score matching, authors found that the mortality rate was 24% in inhalation anesthetics group, which was higher than the mortality rate of 13.6% in propofol group (86). However, this study included multiple tumor surgeries and they did not analyze breast cancer individually. Four systematic reviews and meta-analyses also showed that propofol-based intravenous anesthesia was associated with improved overall survival and recurrence-free survival than volatile anesthesia in all cancer types (91–94). Another two studies from Korea also demonstrated that the effects of total intravenous anesthesia on 5-year overall survival and recurrence-free survival of breast cancer was comparable to that of volatile inhaled anesthesia (87, 88). Similar results were also demonstrated in another 3 retrospective cohort studies from Taiwan (95), Korea (5) and Japan (90). However, a research from Sweden had different results when different statistical adjustment methods were used (89). The overall 5-year survival rate of breast cancer in propofol group was statistically significantly higher than that in the sevoflurane group when statistical adjustments were not applied. However, the 1-year and 5-year survival rates were similar when assessed using propensity score matching. Interestingly, the overall survival in propofol group was again significantly higher after adding study centers in the propensity score matching (89).

**Table 1:** Retrospective clinical studies comparing effects of propofol versus sevoflurane on long-term prognosis of breast cancer.

Country	Cancer	Anesthetic Technique	Number of patients	Evaluations	Outcomes
Swden, 2014 (84)	Breast cancer	Propofol vs. sevoflurane	1837(620 vs. 1217)	1-year and 5-year survival rate	1 year-survival rate: propofol was superior to sevoflurane; 5-year survival rate: no difference
Korea, 2016 (85)	Breast cancer	Propofol vs. sevoflurane	325 (173 vs. 152)	5 year-recurrence-free survival and overall survival	5 year-recurrence-free survival: propofol was superior to sevoflurane; 5 year-overall survival: no difference
UK, 2015 (86)	Mixed cancer	Total intravenous anesthesia (TIVA) vs. volatile inhalational anesthesia (INHA)	7030 (3714 vs. 3316) (2607 in each group after PS matching)	1-yr survival rate and overall mortality rate	TIVA was superior to INHA
Korea, 2017 (87)	Breast cancer	Propofol vs. inhalation anesthetics (sevoflurane, desflurane, isoflurane and enflurane)	2645(56 vs. 2589)	70-monthes recurrence-free survival rate and overall survival rate	Propofol is comparable with volatile agents
Korea, 2019 (88)	Mixed cancer	total intravenous anesthesia (TIVA) vs. volatile inhaled anesthesia (VIA)	729 in each group after PS matching	5-year survival rate	No difference
Korea, 2019 (5)	Breast cancer	IV anesthesia and inhalation anesthesia	7678(3085 vs. 2246); 1766 in each group after PS matching	5-yr recurrence-free survival rates and overall survival	No difference
Sweden, 2020 (89)	Breast cancer	Propofol vs. sevoflurane	6035 (3296 vs. 3209)	1-year survival 5-year survival	Inconsistent conclusions: propofol had higher survival rate without adjusting confounders; No difference in survival by using PS matching; propofol had higher survival rates when adding centers in the PS matching
Japan, 2020 (90)	breast cancer	Propofol vs. sevoflurane	1026(814 vs. 212)	1−year recurrence−free survival	No difference

Despite of large sample size, the inherent defect of retrospective clinical study may contribute to the paradoxical conclusions so far reached. Retrospective studies did not randomize patients to ensure comparable baseline data across groups. In other words, the confounding factors and selection bias are difficult to be controlled in retrospective studies. Furthermore, it is hard to adjust the imbalance between groups in small sample size retrospective studies, for example only 325 patients were included in one study (85). The results from national register-based studies are more accurate due to larger sample size, better precision and the possibility to adjust for more confounders. However, the two recently reported register-based studies from Japan and Denmark compared the difference between propofol and inhalation anesthetics in digestive system neoplasm but not in breast cancer (96, 97). Extremely uneven distribution of population between study groups may also lead to inaccurate results. In a study reported by Kim and colleagues, only 56 patients were included in the propofol group while 2326 patients in inhalation anesthetics group (87). There was only one study that considered the confounding effects of breast cancer subtypes (5), and others ignored the fact that different tumor subtypes may have different responses to anesthetics.

## Randomized Controlled Studies

In order to avoid the shortcomings of retrospective studies and to obtain a more precise causal relationship between general anesthetics and breast cancer outcomes, prospective randomized controlled trials (RCTs) are badly needed. **Table 2** summarized the current RCTs comparing the effects of propofol and sevoflurane on long-term prognosis of breast cancer. A small sample prospective randomized study, conducted in Korea, randomly assigned fifty patients scheduled to receive breast cancer surgery to propofol group and sevoflurane group (34). In this study, the authors evaluated 2 years-recurrence or metastasis. Due to small population, no metastasis was found and only one patient in sevoflurane group had recurrence. Another prospective, randomized and controlled study was conducted in China, which compared the effect of propofol versus sevoflurane on recurrence- free survival rates in 80 breast cancer patients. In this study, the 2-year recurrence- free survival rates had no significant difference between the two groups with 95% in propofol group and 78% in sevoflurane group (p=0.221) (68). Although there was 17% absolute difference, there was no significant difference between these two groups due to relative small sample size. On the basis of their retrospective studies, Enlund et al. designed a RCT to explore the effect of propofol- or sevoflurane- based anesthesia on breast and colorectal cancer (100). The results of 5-year follow up are expected in late 2022. A largest international multi-center RCT to date allocated 2132 breast cancer patients respectively to paravertebral blocks combined propofol group and sevoflurane group. This study showed identical recurrences rate of 10% in either of the two groups, with 3 years median follow-up time (98). However, it is hard to separate the effects of propofol vs. sevoflurane and paravertebral block vs. opioids in the study. Therefore, this study did not conclude propofol or loco-regional anesthesia may impact on cancer outcomes (101). A recent interesting RCT explored the effects of different anesthetics on circulating tumor cells after breast cancer surgery (99). Circulating tumor cells are crucial for tumor metastasis and recurrence (102, 103), and has been confirmed as a promising indicator for prognosis (104). In this study, authors used this indicator to overcome the difficulty of long term follow-up. This study enrolled 210 breast cancer patients in total with 107 patients allocated to sevoflurane anesthesia and 103 patients allocated to propofol anesthesia. The authors found that the median circulating tumor cell counts were similar at 48 hours and 72 hours after surgery between the two groups (99). This study did not compare long-term outcomes of patients, but alternatively examined the effects of propofol and sevoflurane on circulating tumor cell counts, and suggested that these two anesthetics may have similar effect on long-term outcomes of patients with primary breast cancer.

**Table 2:** Randomized controlled trials comparing effects of propofol versus sevoflurane on long-term prognosis of breast cancer.

Country	Cancer	Anesthetic Technique	Number of patients	Evaluations	Outcomes
Korea, 2017 (34)	Breast Cancer	propofol-remifentanil anesthesia and sevoflurane-remifentanil anesthesia	24 patients in each group	NK cell cytotoxicity (NKCC) and 2-year recurrence or metastasis	Propofol anesthesia preserved NKCC; There was no difference in 2-year recurrence or metastasis
China, 2018 (68)	Breast cancer	propofol-remifentanil anesthesia and sevoflurane-remifentanil anesthesia	40 patients in each group	The serum concentrations of VEGF-C and TGF-β before and 24 h after surgery; 2-year recurrence- free survival rate	Sevoflurane increased serum VEGF-C concentrations surgery; There was no difference in 2-year recurrence- free survival rate
International Multi-center, 2019 (98)	Breast cancer	paravertebral blocks combined propofol and sevoflurane with opioid	1043 in paravertebral blocks combined propofol group,1065 in sevoflurane with opioid group	recurrences rate with 36 months median follow-up; Incisional pain at 6 months and 12 months after surgery	The recurrences rate and incisional pain were all comparable between these two groups
Switzerland, 2020 (99)	Primary Breast Cancer	Propofol and sevoflurane anesthesia	103 in propofol and 107 in sevoflurane group	Circulating tumor cell counts at three time points postoperatively (0, 48, and 72 h)	there was no difference between these two groups with respect to circulating tumor cell counts

Other anesthetic drugs and anesthetic techniques are also of concern in breast cancer surgery. Due to the analgesic properties, opioids are widely used during breast cancer surgery. Some laboratory studies showed that opioids inhibit cell-mediated immunity (105), reduce lymphocyte and macrophage proliferation (106), and drive breast cancer metastasis (107). However, the association between opioid-based anesthesia and breast cancer recurrence is inconclusive till now (108). Interestingly, a recent retrospective study with 1143 triple-negative breast cancer (TNBC) cases demonstrated that intraoperative opioids improved the recurrence-free survival of TNBC (109). Local anesthetics have been shown to have the modulatory effects on the immune and inflammatory response, and have antitumor effects, it was hypothesized that regional anesthesia may improve the prognosis of breast cancer. However, there is no high quality clinical evidence to verify these beneficial effects (110). Two studies compared thoracic paravertebral blockade (PVB) with ropivacaine and sham block, in which no difference in breast cancer recurrence rates was found (111, 112).

## Conclusion

Overall, pre-clinical studies and retrospective clinical studies comparing the potential benefits of intravenous propofol over inhalational anesthetics for breast cancer lack consistency. A few current randomized controlled studies suggest that the two anesthetics have similar effects on breast cancer recurrence and metastasis. However, a definite conclusion regarding which anesthetic may have more favorable long-term effects on breast cancer recurrence and metastasis cannot be reached largely due to the lack of multicenter or multi-countries large sample clinical trials.

## Future Research Directions

So far, the effect of different anesthetics or anesthesia techniques on the prognosis of postoperative breast cancer has not been determined. Further investigations should be implemented to explore the mechanisms of anesthetics on breast cancer cells and immune microenvironment. Meanwhile, large sample, multi-center prospective clinical study involving different subtype of breast cancer, different tumor staging should also be conducted. Only a clear understanding of the relationship between anesthetics and breast cancer can improve the prognosis of patients from the perspective of anesthesiologists.

## Author Contributions

YL and PF had the original idea of the manuscript. PF, JZ, and YL reviewed the literature and drafted the article. ZX and XL revised manuscript and provided suggestions for improvement. All authors contributed to the article and approved the submitted version.

## Funding

The author’s study was supported by Advanced Projects of Innovation Program for the Selected Returned Overseas Chinese Scholars, Anhui Province (2020LCX022); National Natural Science Foundation of China (81770295); Bethune Charity Fund project (BCF-RF-WSQZTZJ-202011-057); Young Scholars of Wan Jiang in Anhui Province.

## Conflict of Interest

The authors declare that the research was conducted in the absence of any commercial or financial relationships that could be construed as a potential conflict of interest.

## Publisher’s Note

All claims expressed in this article are solely those of the authors and do not necessarily represent those of their affiliated organizations, or those of the publisher, the editors and the reviewers. Any product that may be evaluated in this article, or claim that may be made by its manufacturer, is not guaranteed or endorsed by the publisher.
